# The formin inhibitor SMIFH2 inhibits members of the myosin superfamily

**DOI:** 10.1242/jcs.253708

**Published:** 2021-04-27

**Authors:** Yukako Nishimura, Shidong Shi, Fang Zhang, Rong Liu, Yasuharu Takagi, Alexander D. Bershadsky, Virgile Viasnoff, James R. Sellers

**Affiliations:** 1 Mechanobiology Institute (MBI), National University of Singapore, Singapore 117411, Singapore; 2 National Heart, Lung, and Blood Institute, National Institutes of Health, Bethesda, MD 20892, USA; 3 Department of Molecular Cell Biology, Weizmann Institute of Science, Rehovot 7610001, Israel; 4 CNRS UMI 3639 BMC, Singapore 117411, Singapore; 5 Department of Biological Sciences, National university of Singapore, Singapore 117558, Singapore

**Keywords:** Formins, Myosins, Actin-activated ATPase, *In vitro* motility assay, Off-target inhibition

## Abstract

The small molecular inhibitor of formin FH2 domains, SMIFH2, is widely used in cell biological studies. It inhibits formin-driven actin polymerization *in vitro*, but not polymerization of pure actin. It is active against several types of formin from different species. Here, we found that SMIFH2 inhibits retrograde flow of myosin 2 filaments and contraction of stress fibers. We further checked the effect of SMIFH2 on non-muscle myosin 2A and skeletal muscle myosin 2 *in vitro*, and found that SMIFH2 inhibits activity of myosin ATPase and the ability to translocate actin filaments in the gliding actin *in vitro* motility assay. Inhibition of non-muscle myosin 2A *in vitro* required a higher concentration of SMIFH2 compared with that needed to inhibit retrograde flow and stress fiber contraction in cells. We also found that SMIFH2 inhibits several other non-muscle myosin types, including bovine myosin 10, *Drosophila* myosin 7a and *Drosophila* myosin 5, more efficiently than it inhibits formins. These off-target inhibitions demand additional careful analysis in each case when solely SMIFH2 is used to probe formin functions.

This article has an associated First Person interview with Yukako Nishimura, joint first author of the paper.

## Abstract (precis)

**Summary:** The inhibitor SMIFH2 inhibits a variety of myosins almost as efficiently as formins, its designated targets.

## INTRODUCTION

Formins are a large and diverse class of actin-associated proteins that is evolutionarily conserved in nature (Breitsprecher and Goode, 2013; Schönichen and Geyer, 2010; van Gisbergen and Bezanilla, 2013). *In vitro*, formin activities include nucleation and elongation of actin filaments (Courtemanche, 2018; Paul and Pollard, 2009; Zigmond, 2004); some formins also bundle actin filaments (Harris et al., 2006; Michelot et al., 2006; Schönichen et al., 2013) and bind to microtubules (Bartolini et al., 2008; Chesarone et al., 2010; Gaillard et al., 2011). Formins contain two types of a characteristic domain, i.e. formin homology (FH) domains 1 and 2. The former domain (FH1) contains proline-rich motifs that interact with the profilin–actin complex, thereby recruiting actin monomers (Courtemanche and Pollard, 2012; Paul et al., 2008). The FH2 domains forms dimers that can nucleate actin filaments and mediate the processive elongation at filament plus-ends, i.e. barbed ends (Aydin et al., 2018; Courtemanche, 2018; Goode and Eck, 2007; Paul and Pollard, 2009). The combined action of FH1 and FH2 domains strongly accelerates filament growth.

Formins are thought to be required for many tasks, including the formation of filopodia, stress fibers, lamellipodia and cytokinetic rings (Breitsprecher and Goode, 2013; Chhabra and Higgs, 2007; Schönichen and Geyer, 2010). However, because of multiplicity of formins – mammals have 15 genes that encode FH1 and FH2 domains – and the apparent redundancy between them, it can be difficult to prove that particular cellular functions depend on formins when using knockout/knockdown experiments. In addition, in some cases, a rapid inhibition of formin function is necessary. Therefore, a broad-specificity formin inhibitor (Rizvi et al., 2009) has been widely used in studies of formin functions *in vivo*.

Rizvi et al. (2009) conducted a small-molecule screen to identify compounds that inhibit the assembly of actin filaments stimulated by the mouse formins mDia1 and mDia2 (officially known as DIAPH1 and DIAPH3, respectively) in the presence of profilin *in vitro*. The compound SMIFH2 was identified to inhibit such assembly in a concentration-dependent manner. Half-maximal inhibition of mDia1 occurred at ∼15 µM SMIFH2 concentration. SMIFH2 did not affect assembly of pure actin. At saturating SMIFH2 concentrations the rate of actin assembly was found to be equal that of actin in the absence of formin (Rizvi et al., 2009), and truncation studies suggested the target domain of the drug to be that of FH2 (Rizvi et al., 2009). Formins from a variety of species, including *C. elegans* (CYK-1), *S. pombe* (Cdc12 and Fus1), *S. cerevisae* (Bn1), and *M. musculus* (mDia2) are also inhibited, with IC_50_ values ranging from 5–15 µM SMIFH2, suggesting that the inhibitor is generally applicable to all formins (Rizvi et al., 2009). This, however, had not been checked directly.

Other inhibitors that affect actin polymerization, such as the marine sponge toxins latrunculin A and B (Spector et al., 1983), jasplakinolide (Bubb et al., 1994), swinholide A (Bubb et al., 1995), fungal cytochalasins (Natori, 1986) or phalloidin (Wieland and Faulstich, 1978) are natural products selected by evolution. High specificity of some of them, e.g. latrunculin A has been confirmed in genetic experiments, showing that yeasts with mutated actin that lacks the ability to bind latrunculin A, are viable, even at very high concentrations of this drug (Ayscough, 1998; Morton et al., 2000). Other toxins, however, can have dual functions, i.e. cytochalasin B that affects both actin polymerization and glucose transport (Kapoor et al., 2016; MacLean-Fletcher and Pollard, 1980; Yamada and Wessells, 1973). By contrast, the chemical structure of SMIFH2 suggests that – due to highly electrophilic nature (Baell, 2010) – this compound is hardly specific, even though molecular targets other than the formin FH2 domain have not been clearly identified yet. Notice that off-target effects have been reported *in vivo*, such as functional alteration of the tumor suppressor protein p53 (TP53), albeit at relatively high concentration of SMIFH2 (Isogai et al., 2015).

Nevertheless, it has been common belief that, at least related to the cytoskeleton and cell motility, this inhibitor can be safely used to identify formin functions, and SMIFH2 has, thus, been widely used in the cytoskeleton community to study formin-dependent actin polymerization in a variety of species, including human, mouse, chicken, zebrafish, *Drosophila*, *Arabidopsis* and yeast, as well as in a number of cell types, including platelets, fibroblasts, epithelial cells and oocytes, and in several cancer cells (Isogai et al., 2015).

Our present study shows that SMIFH2 also appears to be a potent inhibitor of molecular motors of myosin family. During changes of cell motility and shape, actin polymerization does, obviously, function in concert with numerous other processes mediated by certain myosin motors. Thus, any conclusions regarding the involvement of formins – particularly concerning cell functions – that were made solely on the basis of experiments using SMIFH2, should be carefully analyzed and perhaps reconsidered.

## RESULTS

### SMIFH2 inhibits contraction of actomyosin fibers and myosin filament flow in living and permeabilized cells

**Fig. 1.**
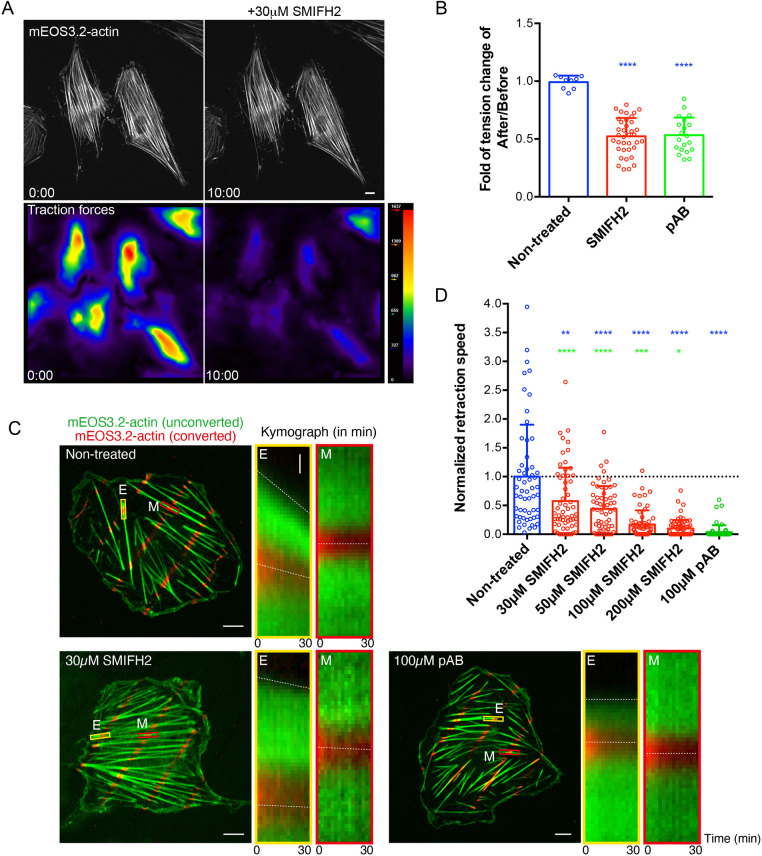
**SMIFH2 inhibits stress fiber contractility in living and permeabilized REF52 cells.** (A) Reduction of traction forces in living REF52 cells after treatment with SMIFH2. Stress fibers were visualized by expressing of mEos3.2-actin (top panels); the magnitude of traction forces exerted by cells on substrate is shown as heat maps (bottom panels) in cells immediately after (left panels) and 10 min after (right panels) the addition of 30 μM SMIFH2. Notice that, although overall actin organization did not change, traction forces dropped dramatically. Scale bar: 10 µm. (B) Quantification of the traction force reduction upon treatment with 30 μM SMIFH2 and 100 μM photo-insensitive para-aminoblebbistatin (pAB); see images of respective experiments in C. Shown are the ratios of mean traction force magnitude per cell 10 min after addition of drug compared with those in the same cells before treatment. *P*-values after comparison of control and respective drug treated groups were calculated using unpaired two-tailed Student’s *t*-test. (C) Experimental set-up as quantified in B. ATP-dependent ventral stress fiber retraction in permeabilized REF52 cells. Cells were visualized by expressing photoconvertible mEOS3.2-actin. Unconverted mEOS3.2-actin is shown in green and pattern of photoconverted mEOS3.2-actin obtained by local laser-illumination is shown in red. Kymographs showing the dynamics of total and photoconverted actin taken at the ends (E, yellow rectangles) or in the middle segments (M, red rectangles) of the ventral stress fibers under each experimental condition. Dashed lines in kymographs indicate movements of the ventral stress fiber ends or photoconverted actin spots. Scale bars: 10 µm. Vertical scale bar in enlarged image of boxed areas: 1 µm. (D) Quantification of the retraction speed of stress fiber ends normalized to the mean speed of non-treated cells (0.025 μm/min). Each dot represents the normalized mean retraction speed of stress fiber ends in one cell. Per cell, ∼40 ends were measured in *n*=56–72 cells under each experimental condition. Bars represent mean±s.d. *P*-values were calculated using two-tailed unpaired Student's *t*-test. Blue asterisks indicate *P*-values after comparison of SMIFH2- or pAB-treated cell groups with the non-treated cell group. Green asterisks indicate *P*-values after comparison of SMIFH2- and pAB-treated cells. *n*>50 cells; ****P<0.0001, ***P<0.001, **P<0.01, *P<0.05; exact *P*-values are shown in Table S1.

The initial observation that triggered this study was the inhibition of traction forces exerted by REF52 fibroblast upon treatment with 30 μM of SMIFH2. The effect was apparent, already within 10 min following addition of SMIFH2, when integrity of the stress fiber system was still well preserved (Fig. 1A). The degree of inhibition of traction forces by SMIFH2 was comparable with that by the myosin 2 ATPase inhibitor para-aminoblebbistatin (pAB) at 100 μM (Fig. 1B).

We further investigated how SMIFH2 would affect the ATP-dependent contractility of linear ventral stress fibers in REF52 cells. To study the effect of SMIFH2 on actomyosin contraction, we used REF52 cells permeabilized with Triton X-100 (Tee and Bershadsky, 2016; Tee et al., 2015; Tint et al., 1991). Detergent treatment leads to depletion of all soluble factors from the cells and, in particular, of ATP. Supplementing the medium with ATP induces the myosin 2-dependent contraction of stress fibers. To monitor the local contraction of the ventral stress fibers at their ends and within the central zone, we expressed photoconvertible mEOS3.2-actin, whose emission wavelength can be converted from green to red channel upon illumination with blue laser (Zhang et al., 2012a). We locally photoconverted spots along the length of ventral stress fibers, and tracked both retraction of the unconverted stress fiber ends and longitudinal movements of the photoconverted actin spots after adding ATP into the solution (Fig. 1C, left panel). We found that ATP addition induced retraction of stress fibers and centripetal displacement of photoconverted actin spots adjacent to their ends. Retraction speed of stress fibers tips was, however, faster than the rate of displacement of photoconverted actin spots nearby (Fig. 1C, middle panel), whereas actin spots in the central zone of stress fibers were hardly mobile (Fig. 1C, right panel). Quantification of retraction speed of stress fiber ends revealed that treatment with SMIFH2 inhibits their ATP-induced retraction in a dose-dependent manner (Fig. 1C and D). At concentrations of >100 μM – i.e. 3-fold more than the typical concentration used in experiments by Rizvi et al. (2009) – we found that treatment with SMIFH2 yields the same level, i.e. complete inhibition, of contractility as does treatment with pAB (Fig. 1D). At concentrations of ∼50μM inhibition is ∼50% reduced, which is still significant when compared to untreated control cells (Fig. 1D).

**Fig. 2.**
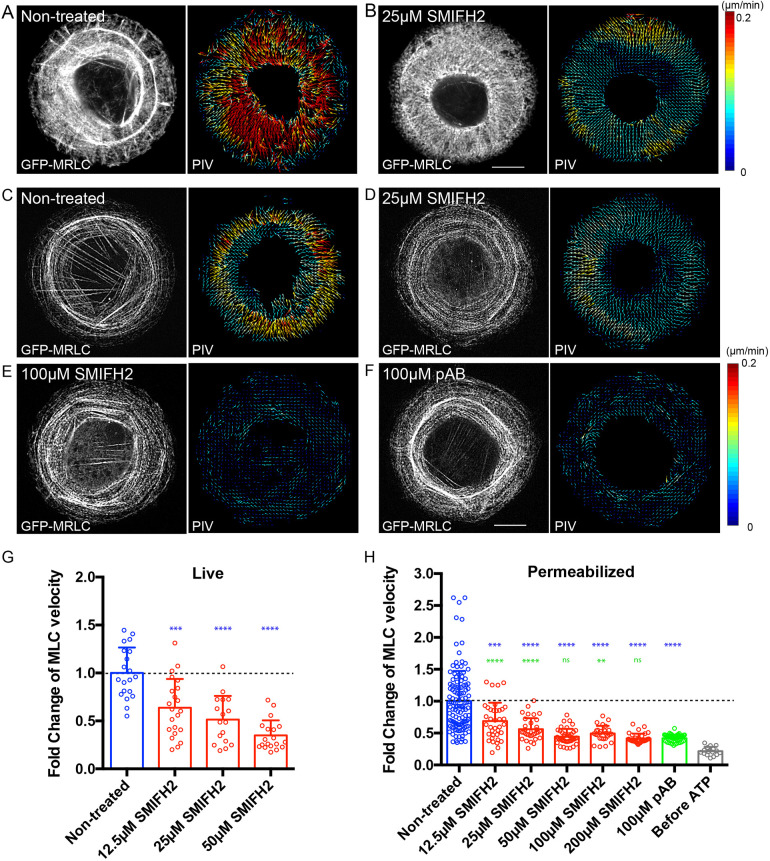
**SMIFH2 inhibits centripetal movement of myosin 2 filaments in living and permeabilized HFF cells.** (A,B) Treatment with SMIFH2 reduces the velocity of centripetal movement of myosin 2 filaments at transverse arcs in the live HFF cells plated on circular fibronectin islands. Shown are representative images of non-treated (A) and SMIFH2-treated (25 μM) cells (B). Myosin 2 mini-filaments were visualized by expression of GFP-MRLC (left panels) and their dynamics were shown as vector maps using particle image velocimetry (PIV) analysis (right panels). Arrows represents direction and velocity with color code shown in the right. Scale bar: 10 µm. (C–F) Effects of SMIFH2 and photo-insensitive para-aminoblebbistatin (pAB) on the velocity of centripetal movement of myosin 2 filaments induced by ATP in permeabilized HFF cells. Representative images of myosin 2 filaments (GFP-MRLC, left) and their dynamics (PIV, right) are shown in non-treated cells (C), in cells treated with SMIFH2 at 25 μM (D) or 100 μM (E), and in cells treated with pAB at 100 μM (F). Scale bar: 10 µm. (G) Quantification of the velocity of myosin 2 filament in non-treated and SMIFH2-treated (12.5, 25 or 50 μM) living cells. Treatment with SMIFH2 reduces the centripetal movement of myosin 2 filament in a dose-dependent manner. Bars represent the mean±s.d. and each dot represents the value of PIV per cell (*n*≥17 cells). Values were normalized to the mean speed in non-treated cells (0.152 μm/min). (H) Quantification of the ATP-dependent velocity of myosin 2 filaments in permeabilized cells with or without pharmacological perturbation. Bars represent the mean±s.d. and each dot represents the mean value of PIV per cell (*n*≥16 cells). Values were normalized to the mean velocity in non-treated cells (0.0745 μm/min). *P*-values were calculated using two-tailed unpaired Student’s *t*-test. Blue asterisks indicate *P*-values after comparison of SMIFH2- or pAB-treated cells with the non-treated group of cells. Green asterisks indicate *P*-values after comparison of SMIFH2- and pAB-treated cells. ****P<0.0001, ***P<0.001, **P<0.01, *P<0.05; exact *P*-values are shown in Table S1.

We also tested inhibition of contraction of another type of contractile actomyosin structure, i.e. of the transverse arcs formed by periodically arranged myosin and actin filaments in fibroblasts (Hu et al., 2017). We measured the velocity of movement of transverse arcs in human foreskin fibroblast (HFF) cells plated on a circular fibronectin island (Tee et al., 2015). In control cells, filaments of non-muscle myosin 2 were visualized by expression of GFP-tagged myosin regulatory light chain (GFP-MRLC) localized to the transverse arcs and moved towards the cell center with an average velocity of 0.152 μm/min, as determined by particle image velocimetry (PIV) (Fig. 2A) (Hu et al., 2017). Here, we showed that the velocity of this movement decreased in dose-dependent manner when cells were treated with SMIFH2 for 45 min (Fig. 2B; Fig. 2G). However, such SMIFH2 treatment also affected the overall organization of myosin 2 filaments within cells (Fig. 2B), which is in agreement with previous publications (Tee et al., 2015). Permeabilization of the same cells by using Triton X-100 removed G-actin and ATP. Centripetal movement of transverse arcs in permeabilized cells can be induced by addition of ATP to the incubation buffer (Tee and Bershadsky, 2016; Tee et al., 2015). Treatment with SMIFH2 inhibited ATP-induced centripetal movement of myosin 2 filaments in permeabilized cells (Fig. 2C,D and H). At a concentration of 50 μM, SMIFH2 blocked centripetal movement as efficient as pAB (Fig. 2E,F and H).

The above-described effects SMIFH2 has on stress fiber retraction and actin arc movement in permeabilized cells cast some doubt on the inhibitory selectivity of SMIFH2 regarding formin-dependent actin polymerization. Indeed, permeabilized cells do not contain G-actin, and incubation buffer was supplemented with phalloidin, which stabilizes the actin filaments. Thus, the process of actin filament polymerization or depolymerization is hardly possible within this system. Given that addition of SMIFH2 phenocopied the action of pAB in our assay, raised the question whether SMIFH2 is also able to inhibit paralogs of non-muscle myosin 2. To address this, we examined the effect of SMIFH2 on myosins *in vitro*.

### Effects of SMIFH2 on myosins *in vitro*

**Fig. 3.**
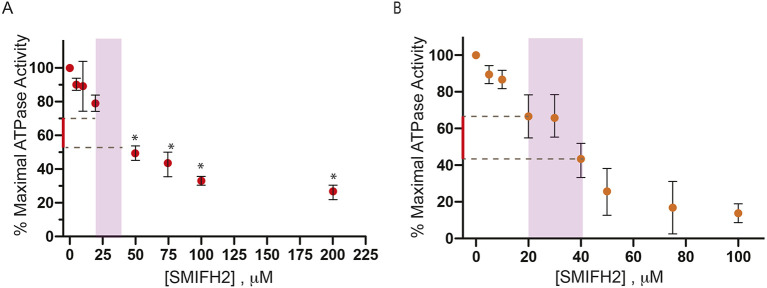
**Inhibition of myosin 2 activity by SMIFH2.** Inhibition of (A) actin-activated ATPase of human non-muscle myosin 2A and (B) rabbit skeletal muscle myosin 2 by SMIFH2. Error bars denote ±s.d. of at least three independent assays. *P<0.05. Purple areas indicate the typical concentration range of SMIFH2 as used in the majority of publications. Parts of the *y*-axes in red denote the extent of inhibition that can be approached when SMIFH2 concentrations do not exceed these values.

Two methods are primarily used to assess actomyosin function *in vitro*. These are the actin-activated ATPase assay analyzing the interaction between myosin and its inhibitor SMIFH2, and the gliding actin *in vitro* motility assay that assesses the ability of myosin to propel actin filaments (see Materials and Methods). In the absence of actin, myosins have very low basal ATPase rates that are activated 10–1000-fold by the addition of actin (De La Cruz and Ostap, 2009). In some cases, we also used soluble fragments of myosin, termed heavy meromyosin (HMM), for these assays, which are excellent to model the behavior of intact myosin but do not form filaments – a fact that complicates the measurement of ATPase activity *in vitro*. We first investigated the effect SMIFH2 might have on actin-activated magnesium-dependent ATPase (MgATPase) activity of human non-muscle myosin 2A (heavy chain encoded by *MYH9*), whose enzymatic activity depends on phosphorylation of its regulatory light chain subunit through myosin light chain kinase (MLCK). We found that SMIFH2 does, indeed, inhibit activity of this myosin in a dose-dependent manner, with an IC_50_ of ∼50 µM (Fig. 3A). Therefore, if SMIFH2 also were to inhibit the activity of MLCK, treatment of cells with this drug would effectively prevent activation of the pool of non-muscle myosin 2A that is normally activated by this phosphorylation. We found that SMIFH2 did not inhibit activity of MLCK – which was used to phosphorylate non-muscle myosin 2A (Table S2). SMIFH2 also inhibited the actin-activated ATPase activity of rabbit skeletal muscle myosin 2 (tissue purified, mixed isoforms) with an IC_50_ of ∼40 µM (Fig. 3B). At a concentration of 100 µM, SMIFH2 reduced the basal ATPase activity in the absence of actin by 73% (from 0.36 s^−1^ down to 0.009 s^−1^; average of two experiments), demonstrating that the drug targets myosin rather than indirectly inhibiting it via binding to actin.

We next examined the effect of SMIFH2 on the ability of skeletal muscle myosin to translocate actin, by using a gliding actin *in vitro* motility assay. Here, skeletal muscle myosin 2 HMM is bound to a nitrocellulose-coated coverslip and its ability to translocate Rhodamine–phalloidin-labeled actin filaments observed (Table 1). In the absence of SMIFH2>90% of actin filaments were motile and moved with a velocity of 5.4±0.08 μm s^−1^ (mean±s.e.m.). This activity was completely abolished at a SMIFH2 concentration of 150 µM and could not be reversed, even by extensive washout using motility buffer without SMIFH2. The lack of reversibility could be due to covalent modification of myosin by SMIFH2. Interestingly, under these conditions, immobile actin filaments were tethered to the surface but fewer filaments were present. This was in contrast to conditions of without or reduced concentrations of SMIFH2. At intermediate concentrations of SMIFH2 (50 and 100 µM) average velocity was reduced (Table 1) but the number of motile filaments still remained high. A higher drug concentration was required to achieve ∼50% of the gliding velocity than was required for similar inhibition of the actin-activated ATPase. This was also observed when blebbistatin was used to inhibit actin gliding (Limouze et al., 2004; Sakamoto et al., 2005). This, together with the observation that fewer surface-bound actin filaments were observed at saturating SMIFH2 concentration (>150 µM), suggests that the drug blocks the myosin kinetics cycle when in a weakly bound state, similar to what was observed for inhibition of myosin 2 isoforms in response to blebbistatin (Kovács et al., 2004; Ramamurthy et al., 2004). Thus, the differences in the concentration of SMIFH2 required to inhibit ATPase activity and *in vitro* translocation of actin can be explained. The level of inhibition of ATPase activity is the numerical average of the number of myosin molecules bound by SMIFH2 – i.e. totally inhibited, and of the number of myosins molecules that are not drug bound – i.e. maximally activated. When 50% of myosin is bound by SMIFH2, the observed level of actin-activated ATPase activity is halved. By contrast, in the gliding actin *in vitro* motility assay, the rate of actin filament sliding does not depend strongly on the number of myosin molecules that contribute to movement, i.e. the myosin-surface density. Therefore, when 50% of myosin is bound by SMIFH2, the amount of active myosin on the surface is still sufficient to propel actin filaments at full velocity. However, if binding to SMIFH2 blocks the kinetics cycle of myosin to create molecules that can weakly – but not productively – bind to actin, these weakly bound myosins exert a small frictional drag on the actin filament that will slightly inhibit its velocity (Table 1). This, along with the weakly tethered actin filaments observed on the surface when the concentration of SMIFH2 is saturated, suggest that SMIFH2 – in way similar to the kinetics of blebbistatin – blocks the release of phosphate from the actomyosin–ADP–*P*i complex, which can only weakly bind to actin and cannot complete the powerstroke.

**Table 1.**
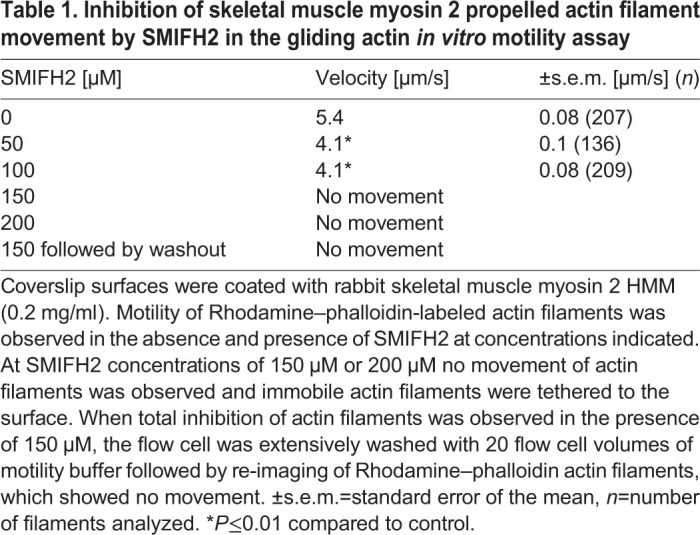
Inhibition of skeletal muscle myosin 2 propelled actin filament movement by SMIFH2 in the gliding actin *in vitro* motility assay

SMIFH2 also inhibited the movement of actin filaments propelled by phosphorylated non-muscle myosin 2A (Table 2). Not surprisingly, higher concentrations of SMIFH2 were required and only partial inhibition was obtained at 250 µM SMIFH2.

**Table 2.**
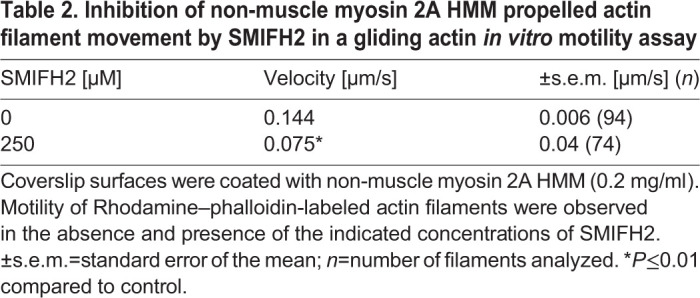
Inhibition of non-muscle myosin 2A HMM propelled actin filament movement by SMIFH2 in a gliding actin *in vitro* motility assay

**Fig. 4.**
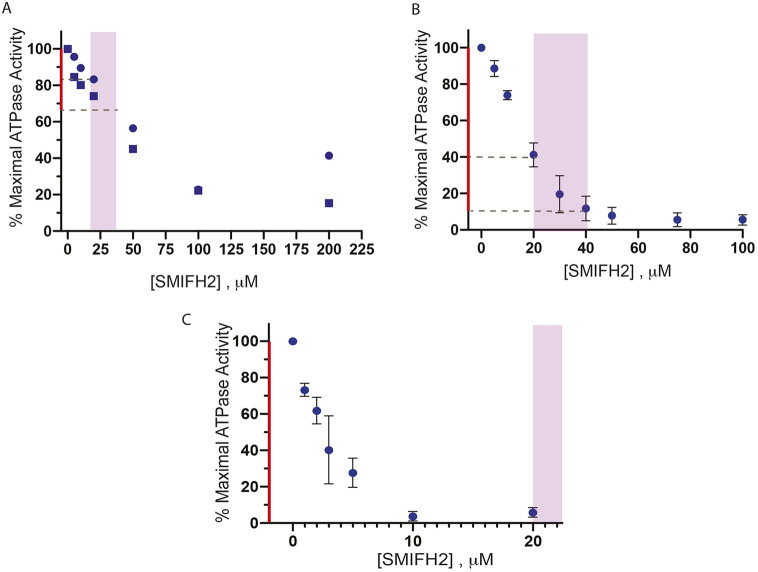
**Inhibition of non-conventional myosins by SMIFH2.** (A) Inhibition of the actin-activated ATPase activity of *Drosophila* myosin 7a. Duplicate measurements are indicated by squares and dots. (B) Inhibition of actin-activated ATPase activity of bovine myosin 10. (C) Inhibition of actin-activated ATPase activity of *Drosophila* myosin 5. Purple areas indicate the typical concentration range of SMIFH2 as used in the majority of publications. Parts of the *y*-axes in red denote the extent of inhibition that can be approached when SMIFH2 concentrations do not exceed these values. Error bars in B and C denote ±s.d. of least three independent assays.

There are 40 myosin heavy chain genes from twelve classes in the human genome (Berg et al., 2001). Many, but not all of these myosins are present in other metazoan species including *Drosophila* (Yamashita et al., 2000). Although all of these myosins contain a conserved motor domain, the tail portions are very diverse, allowing the myosins to perform a plethora of cellular functions. Blebbistatin was shown to specifically inhibit class 2 myosins (Limouze et al., 2004). To determine whether SMIFH2 inhibits other myosin classes, we assayed its effect on *Drosophila* myosin 5 (heavy chain encoded by *didum*), *Drosophila* myosin 7a (heavy chain encoded by *crinkled*) and bovine myosin 10 (heavy chain encoded by *MYO10*) (summarized in Table S3). SMIFH2 inhibited the ATPase activity of each of these myosins with various degrees of potency, i.e. *Drosophila* myosin 7a with an IC_50_ of ∼30 µM (Fig. 4A), bovine myosin 10 with an IC_50_ of ∼15 µM (Fig. 4B) and, interestingly, *Drosophila* myosin 5 with an IC_50_ of ∼2 µM (Fig. 4C). Thus, SMIFH2 inhibits *Drosophila* myosin 5 even more potently than it does formins *in vitro*. The assays regarding bovine myosin 10 are particularly interesting since this myosin plays a role in filopodia initiation and formation in mammalian cells (Kerber and Cheney, 2011), a process that also involves formin action. The sensitivity of bovine myosin 10 to SMIFH2 does, therefore, question the use of this drug in studies of filopodia formation.

## DISCUSSION

The actin cytoskeleton of cells consists of many distinct higher order arrays of actin filaments, such as the branching network found in the lamellipodia, transverse arc bundles, ventral and dorsal stress fibers, and filopodia (Blanchoin et al., 2014; Svitkina, 2018). Some cells contain specialized actin arrays, such as microvilli within intestinal enterocytes and stereocilia in the hair cells of the ear (Pelaseyed and Bretscher, 2018). These arrays are formed by actin nucleators, such as the Arp2/3 complex, formins and several others, with the assistance of a variety of actin-associated proteins (Courtemanche, 2018; Merino et al., 2020; Rottner et al., 2017; Siton-Mendelson and Bernheim-Groswasser, 2017; Swaney and Li, 2016). SMIFH2 was discovered in a small-molecule screen for compounds that inhibit formin-driven actin polymerization *in vitro* but do not affect polymerization of pure actin (Rizvi et al., 2009). Numerous studies describe the use of SMIFH2 to determine the involvement of formins in cellular and developmental processes, and to investigate any immediate effects of formin inhibition. SMIFH2 has been used at concentrations ranging from 5–100 µM and for incubation times of <1 h to >24 h.

We found here that a moderate SMIFH2 concentration (30 μM) very rapidly reduces the traction forces exerted by fibroblasts on their substrates. Further, by using cells confined to micropatterned circular adhesive islands, we demonstrated that SMIFH2 inhibits the retrograde flow of myosin filaments in the course of centripetal movement of contracting transverse arcs. By themselves, these findings were not alarming since the dynamics of the actin polymerization /depolymerization and interaction of myosins with actin are thought to be tightly linked (Agarwal and Zaidel-Bar, 2019; Zimmermann et al., 2015). However, the fact that SMIFH2 inhibited the ATP-dependent movement of transverse arcs and the retraction of ventral stress fibers in permeabilized cells in a manner resembling the blebbistatin inhibition of these processes was unexpected. These permeabilized cells do not contain G-actin and their actin cytoskeleton is stabilized with phalloidin, which makes the processes of actin polymerization/depolymerization near impossible. This led us to investigate the possibility that SMIFH2 also inhibit non-muscle myosin 2A.

Our results showed that SMIFH2 does, indeed, inhibit the actin-activated ATPase activity of non-muscle myosin 2A. Moreover, since SMIFH2 did not inhibit MLCK-mediated phosphorylation of myosin 2A light chain – which is required for activation of non-muscle myosin 2A – this was a direct effect on the activity of the myosin. SMIFH2 also inhibited both the actin-dependent and the basal, i.e. in the absence of actin, activity of skeletal muscle myosin 2. *In vitro*, gliding of actin filaments due to myosin 2A was also suppressed by SMIFH2. Thus, direct inhibition of myosin 2A by SMIFH2 could be involved in the effects of SMIFH2 observed in cells. In our experiments, the concentrations of SMIFH2 sufficient to stop the flow of non-muscle myosin 2A filaments in living and even permeabilized cells were, however, still below the concentrations needed to substantially inhibit non-muscle myosin 2A function *in vitro*. Of note, the effect of high-dose (150 µM) SMIFH2 on gliding of actin filaments via skeletal non-muscle myosin 2 was irreversible, whereas effects of lower doses of SMIFH2 were shown to be reversible *in vivo* (Tee et al., 2015). We have recently shown that SMIFH2 can efficiently detach actin filaments from mDia1 formin anchored to the glass surface of a microfluidic chamber (Alieva et al., 2019). This suggest that some effects of SMIFH2 *in vivo* and even in permeabilized cells are, at least partially, explained by disruption of the actin network connectivity. This possibility deserves further investigation. Nevertheless, non-muscle myosin 2 paralogs, i.e. non-muscle myosins 2A, 2B and 2C, are involved in many cellular processes that are also thought to involve formins, such as platelets formation (Pal et al., 2020; Pan et al., 2014; Zhang et al., 2012b), assembly of the cytokinetic contractile ring (Pollard and O'Shaughnessy, 2019; Taneja et al., 2020) and maintenance of stress fiber integrity (Hu et al., 2017; Oakes et al., 2012). Thus, any conclusions regarding formin function solely the basis of experiments that used SMIFH2 should be carefully revisited.

Even more surprising were our findings that SMIFH2 affects some other types of myosin to a greater extent than myosin 2A and, in some cases, more than it affects formins. We have demonstrated that SMIFH2 inhibits myosins from all classes tested, including rabbit skeletal muscle myosin, *Drosophila* myosins 5 and 7a and bovine myosin 10. The IC_50_ values for inhibition of myosin 10 were similar to that of formin inhibition. The SMIFH2 IC_50_ for *Drosophila* myosin 5 was ∼2 µM, meaning that SMIFH2 is more potent than published SMIFH2 IC_50_ values for a variety of formins (Rizvi et al., 2009).

The actions of formins and myosin are intimately linked in cells since most of the actin arrays built or influenced by formins interact with myosins. For example, formation of the cytokinetic ring depends on formins (Pollard and O'Shaughnessy, 2019) but non-muscle myosin 2 paralogs are essential for its function (Taneja et al., 2020; Yamamoto et al., 2019). Filopodia elongation has been proven to be formin-dependent in formin knockdown and overexpression experiments (Mellor, 2010; Schaks et al., 2019) but many studies have demonstrated that myosin 10 also plays an important role. Myosin 10 has been found in the patches at the tips of filopodia within mammalian cells and its knockdown in a variety of cells is associated with filopodia suppression (Arjonen et al., 2011; Kerber and Cheney, 2011). In addition, non-muscle myosin 2A was shown to play a role in the stabilization of filopodia adhesion (Alieva et al., 2019). In our recent experiments, effect of SMIFH2 on filopodia involves disintegration of myosin 10 patches at filopodia tips and the myosin 2A-dependent centripetal movement of residual myosin 10 puncta along filopodia (Alieva et al., 2019). Thus, SMIFH2 treatment did not inhibit myosin 2A activity in these experiments but its effect on myosin 10 cannot be excluded. Therefore, experiments using SMIFH2 alone do not permit to dissect the functions of formins and myosins in filopodia.

Some mutations in mDIA1 correlate with hearing loss, and there is some evidence of formin involvement regarding the formation of stereocilia in specialized inner ear cells (Neuhaus et al., 2017; Ueyama et al., 2016). At the same time, the formation and function of these same structures depends on several myosin isoforms, including myosin 1C (Stauffer et al., 2005), myosins 3a and b (Lelli et al., 2016), myosin 6 (Hertzano et al., 2008; Seiler et al., 2004), myosins 7a (Morgan et al., 2016; Yu et al., 2017), and myosin 15 (Anderson et al., 2000; Friedman et al., 1999). Formation and maintenance of actin bundle integrity – i.e. of radial/dorsal fibers, transverse arcs and ventral stress fibers – depend on formins (Hotulainen and Lappalainen, 2006; Oakes et al., 2012; Schulze et al., 2014); however, these structures also depend on the generation of force driven by myosin 2, and paralogs of non-muscle myosin 2 are components of some of these structures (Beach et al., 2014; Hu et al., 2017; Kuragano et al., 2018; Shutova et al., 2017; Vicente-Manzanares et al., 2009). Myosin 2 and 7a also have conserved functions in cell adhesion (Küssel-Andermann et al., 2000; Titus, 2005; Velichkova et al., 2002; Vicente-Manzanares et al., 2009), a process in which formins are also participating (Grikscheit and Grosse, 2016; Romero et al., 2020). Thus, it would be difficult to interpret studies of formin functions by using a compound that inhibits formins as well as myosins.

An unsolved question is whether there is some structural similarity between myosins and formins, which could explain the dual specificity of SMIFH2. Such similarity, however, does not necessary exist since the highly electrophilic nature of SMIFH2 makes this compound very promiscuous in its interactions with different proteins, as has been recognized in screening-based studies (Baell, 2010).

In summary, our study demonstrates that SMIFH2 can no longer be considered to be a specific inhibitor of formins in studies investigating cell motility and actomyosin cytoskeleton organization. Conclusions based on using SMIFH2 in such studies should be carefully reconsidered and, possibly, reinterpreted. The development of novel more-specific inhibitors suitable for instant suppression of formin functions in cells is, therefore, an important and timely task for future studies.

## MATERIALS AND METHODS

### Cell culture and transfection

Immortalized rat embryo fibroblasts (REF52 cells) cell line (Matsumura et al., 1983) and human foreskin fibroblasts (HFFs) (American Type Culture Collection, Manassas, VA, USA; catalogue no. SCRC-1041) were cultured in Dulbecco's modified Eagle's medium (DMEM; Invitrogen, 11965092) supplemented with 10% heat-inactivated fetal bovine serum (FBS; Invitrogen, 10082147) and 1% penicillin/streptomycin (Invitrogen, 15070063) at 37°C and 5% CO_2_. Both cell lines were regularly tested for mycoplasma contamination by using the MycoAlert PLUS Mycoplasma Detection Kit (Lonza, LT07-703). REF52 cells were transiently transfected with the mEos3.2-Actin expression vector (Michael W. Davidson group collection, The Florida State University, Tallahassee, FL, USA; kindly provided by Dr P. Kanchanawong, MBI, Singapore; Addgene 57443) by using jetPRIME transfection reagent (Polyplus transfection, 114-15) in accordance with the manufacturer's protocols. HFF cells were transfected with GFP-tagged myosin regulatory light chain (GFP-MRLC) expression vector (Kengyel et al., 2010) (a gift from W. A. Wolf and R. L. Chisholm, Center for Genetic Medicine, Feinberg School of Medicine, Northwestern University, Chicago, IL, USA) using electroporation (Neon transfection system, Life Technologies) following the manufacturer's instructions.

### Traction force microscopy

Traction force microscopy with embedded beads was performed as described previously (Rafiq et al., 2019). Briefly, soft polydimethylsiloxanes CY 52-276A and CY 52-276B (Dow Corning, 0008602722) were mixed at the ratio 1:1 and Sylgard 184 crosslinker was used to tune the stiffness of the gel for proper force measurement of cells (∼95 kPa). The mixture was spin-coated onto a clean coverslip to achieve the thickness of ∼7 μm and cured for 1 h at 80°C. The surface of the gel was silanized with (3-aminopropyl)triethoxysilane for 2 h, followed by incubation of 0.04 μm FluoSpheres carboxylate-modified microspheres, dark red fluorescent (660/680) beads (Thermo Fisher Scientific, 1871942) at 1×10^6^ beads/ml in a solution of 0.1 M NaHCO_3_ for 30 min. Before seeding the cells, the coverslips with beads were further incubated for 30 min with 10 μg/ml fibronectin, also dissolved in 0.1 M NaHCO_3_. Traction forces were calculated from bead displacement fields visualized by using live cell imaging as described by Tseng et al. (2012), and using the online ImageJ plugin (https://sites.google.com/site/qingzongtseng/tfm for plugin software details). The computation algorithm as published by Sabass et al. (2008) was used. The distribution of traction force magnitude was presented as a heat map (Fig. 1A), and mean magnitude values were calculated for each cell.

### Experiments with Triton X-100-permeabilized cells

For HFF cells, circular adhesive islands of fibronectin were fabricated by stencil patterning as described previously (Jalal et al., 2019). GFP-MRLC expressing HFF cells were then seeded at density of 5×10^4^ cells/ml on the hydrophobic uncoated μ-dish 35 mm (ibidi, 81151) with fibronectin micro-patterns and incubated 3-8 h prior to the experiment. For stress fiber imaging, REF52 cells were transfected with mEos3.2-Actin expression plasmid and seeded onto a 35 mm glass bottom dish (Iwaki, 3930-035) 24 h prior to the assay.

The protocol of the cell permeabilization and cytoskeleton contractility assay has been described previously (Tee and Bershadsky, 2016; Tee et al., 2015). Briefly, cells were permeabilized with extraction buffer A [50 mM imidazole pH 6.8, 50 mM KCl, 0.5 mM MgCl_2_, 0.1 mM EDTA, 1 mM EGTA, 1 mM 2-mercaptoethanol, 250 nM phalloidin (Thermo Fisher Scientific, P34572) and 2 μg/ml protease inhibitor cocktail (Sigma-Aldrich, P8340)] supplemented with 0.1% Triton X-100 and 4% PEG MW35000 for 10 min at room temperature, followed by three washes with extraction buffer A. Cytoskeleton contractility assay was carried out at 37°C using buffer A supplemented with 2 mM ATP with or without the appropriate drugs, i.e. SMIFH2 (Sigma-Aldrich, S4826) or para-aminoblebbistatin (pAB, Optopharma, DR-Am-89). All drug remained in the buffer during the entire period of observation.

### Live cell imaging and confocal microscopy

Super-resolution SIM imaging was performed by using a W1-spinning-disc confocal unit coupled with the live super-resolution (SR) module (spinning-disk-based structured illumination super resolution (York et al., 2013), GatacaSystems), mounted on an Eclipse microscope with Perfect Focus System, supplemented with Plan Apo 100× oil NA1.45 or 60×1.20 NA CFI Plan Apo Lambda water immersion (Nikon) and scientific complementary metal–oxide–semiconductor (sCMOS) camera Prime95B (Photometrics) objectives. Laser lines wavelengths at 488, 561 and 647 nm were used. For HFF cells, time-lapse images at 2 min intervals of *z*-stacks, step-size 0.35 μm were acquired. For REF52 cells, time-lapse images were acquired for 30 min at 5 min intervals at the basal plane of cells.

### Image analysis

Particle Image Velocimetry (PIV) analysis was used to measure average instantaneous speed of filaments of non-muscle myosin 2 visualized by expression of GFP-MRLC. PIV analysis was performed using MatPIV 1.6.1. Single-pass PIV with a window size of 32×32 pixels and 50% overlap was applied. The average instantaneous speed for the first two frames within the region of interest was computed. To quantify the retraction speed of stress fiber ends, we manually selected all ends labeled by unconverted mEOS3.2-actin and arranged them into kymographs for every cell. The retraction speed for each end during imaging was calculated manually using the kymograph. The average retraction speed of stress fibers was calculated for each cell and plotted.

### Statistical analyses

Plotting and statistical analysis were done by using GraphPad Prism 7 (GraphPad Software). Significant differences (*P*-value) were calculated using two-tailed unpaired Student's *t*-test. Bar graphs and scatter plots show mean±s.d. for the respective groups of data.

### Preparation of proteins

A heavy meromyosin (HMM)-like fragment of human non-muscle myosin 2A was prepared by expression in Sf9 insect cells as described (Kengyel et al., 2010). Cells were co-infected with a virus driving the expression of the truncated myosin heavy chain as well as one driving the expression of regulatory and essential light chains. The myosin was phosphorylated through smooth muscle myosin light chain kinase (MLCK) prior to use (Nagy et al., 2013). Full-length *Drosophila* myosin 5 (Lu et al., 2020), *Drosophila* myosin 7a (Yang et al., 2009) and a forced dimeric HMM-like fragment of bovine myosin 10 (Takagi et al., 2014) were also produced in Sf9 cells. *Drosophila* myosin 5 was co-expressed with *Drosophila* ELC and calmodulin, whereas the HMM-like fragment of myosin 10 was co-expressed with calmodulin. Skeletal muscle HMM was produced by chymotrypic digestion of full-length rabbit fast skeletal muscle myosin (Margossian and Lowey, 1982). Rabbit skeletal muscle actin was prepared as previously described (Lehrer and Kerwar, 1972).

### Actin-activated ATPase assays

Actin-activated ATPase assays were carried out using an NADH-linked assay in a Cary 50 spectrophotometer as previously described (Heissler et al., 2015). SMIFH2 was first prepared as a 50 mM solution in DMSO and diluted in DMSO as required. The final amount of DMSO added to the samples was 2%. The assay conditions were 10 µM actin, 50 mM KCl, 10 mM MOPS, 2 mM MgCl_2_, 0.1 mM EGTA, 1 mM ATP, 10 mM MOPS pH 7.0 at 25°C. The buffer also contained 40 units/ml l-lactic dehydrogenase, 200 units/ml pyruvate kinase, 200 μm NADH, and 1 mm phosphoenolpyruvate. Absorbance was monitored at 340 nm. Non-muscle myosin 2A was first phosphorylated by incubation in 0.3 M KCl, 4 mM MgCl_2_, 0.2 mM CaCl_2_, 0.1 mM EGTA, 0.1 mM ATP, 1 μM calmodulin, 2 nM MLCK for 10 min at room temperature.

### Gliding actin *in vitro* motility assay

The gliding actin *in vitro* motility assay was conducted at 30°C in 50 mM KCl, 4 mM MgCl_2_, 0.1 mM EGTA, 30 mM MOPS pH 7.2, 0.5% methylcellulose, 1 mM ATP, using an oxygen-savaging system consisting of 2.5 μg/ml glucose oxidase, 45 μg/ml catalase, 2.5 mg/ml glucose and 50 mm DTT (Sellers et al., 1993). The rate of movement of actin filaments was determined as described previously (Homsher et al., 1992).

### HPLC-mass spectrometry for protein phosphorylation

Phosphorylation of non-muscle myosin 2A was initiated by the addition of ATP. Samples were taken at different time points and diluted with 305 acetonitrile, 0.25 TFA to stop the reaction. Proteins were injected into a reverse phase HPLC (Agilent 1100 series HPLC, Agilent Technologies) with a Zorbax 300SB-C18 (2.1×50 mm, 3.5 mm, Agilent Technologies) and introduced into the mass spectrometer as described (Apffel et al., 1995; Taggart et al., 2000). Positive ion Electrospray Ionization (ESI) mass spectra for intact protein were obtained with an Agilent 6224 mass spectrometer equipped with an ESI interface and a time-of-flight (TOF) mass detector (Agilent Technologies). Mass spectra were analyzed and de-convoluted using a software, MassHunter version B.06.00 (Agilent Technologies).

## Supplementary Material

Supplementary information

Reviewer comments
