# Supplementary material for: The formin inhibitor SMIFH2 inhibits members of the myosin superfamily
Source: J Cell Sci. 2021 Apr 27;134(8):jcs253708. doi: 10.1242/jcs.253708 (PMC8121067; doi:10.1242/jcs.253708)
Supplement: Supplementary information [file joces-134-253708-s1.pdf]

Table S1

(1) P values in Figure 1B

	SMIFH2	pAB
Non-treated	< 0.0001	< 0.0001

(2) P values in Figure 1D

	30µM SMIFH2	50µM SMIFH2	100µM SMIFH2	200µM SMIFH2	100µM pAB
Non-treated	0.0030	< 0.0001	< 0.0001	< 0.0001	< 0.0001
100µM pAB	< 0.0001	< 0.0001	0.0003	0.0107	

(3) P values in Figure 2G

	12.5µM SMIFH2	25µM SMIFH2	50µM SMIFH2
Non-treated	0.0002	< 0.0001	< 0.0001

(4) P values in Figure 2H

	12.5µM SMIFH2	25µM SMIFH2	50µM SMIFH2	100µM SMIFH2	100µM SMIFH2	100µM pAB
Non-treated	0.0002	< 0.0001	< 0.0001	< 0.0001	< 0.0001	< 0.0001
100µM pAB	< 0.0001	< 0.0001	0.2153	0.0009	0.7841	

Table S2

	0 µM SMIFH2	100 µM SMIFH2
**Time, min.**	**% phosphorylated**	**% phosphorylated**
0.5	50.8	55.4
1.5	71.2	70.4
4.5	82.1	81.2

Table S2: SMIFH2 has no effect on myosin light chain kinase. Nonmuscle myosin 2A was incubated with myosin light chain kinase in the presence or absence of 100 µM SMIFH2 for the indicated times. The extent of phosphorylation was quantified by mass spectrometry (Apffel et al., 1995; Taggart et al., 2000).

Table S3

Myosin	IC_{50} (µM SMIFH2)
**Human non-muscle Myo2A**	**50**
**Rabbit skeletal muscle Myo2**	**30**
**Drosophila Myo7A**	**30**
**Bovine Myo10**	**15**
**Drosophila Myo5**	**2**

Table S3: Recapitulation of IC_{50} for the various myosin types we probed in this work
