# Supplementary material for: The formin inhibitor SMIFH2 inhibits members of the myosin superfamily
Source: J Cell Sci. 2021 Apr 27;134(8):jcs253708. doi: 10.1242/jcs.253708 (PMC8121067; doi:10.1242/jcs.253708)
Supplement: Reviewer comments [file joces-134-253708_review_history.pdf]

The formin inhibitor SMIFH2 inhibits members of the myosin superfamily

Yukako Nishimura, Shidong Shi, Fang Zhang, Rong Liu, Yasuharu Takagi, Alexander D. Bershadsky, Virgile Viasnoff and James R. Sellers
DOI: 10.1242/jcs.253708

Editor: Michael Way

Review timeline
Original submission: 23 September 2020
Editorial decision: 26 October 2020
First revision received: 31 January 2021
Accepted: 3 February 2021

Original submission

First decision letter

MS ID#: JOCES/2020/253708

MS TITLE: The Formin Inhibitor, SMIFH2, Inhibits Members of the Myosin Superfamily

AUTHORS: Yukako Nishimura, Shidong Shi, Fang Zhang, Rong Liu, Yasuharu Takagi, Alexander D. Bershadsky, virgile viasnoff, and James R. Sellers

ARTICLE TYPE: Research Article

We have now reached a decision on the above manuscript.

To see the reviewers' reports and a copy of this decision letter, please go to: https://submit-jcs.biologists.org and click on the 'Manuscripts with Decisions' queue in the Author Area. (Corresponding author only has access to reviews.)

As you will see, all three reviewers gave favourable reports but raised some points that will require amendments to your manuscript including resolving the potency of SMIFH2 for Non-muscle Myosin2A. I hope that you will be able to carry these out, because I would like to be able to accept your paper.

We are aware that you may be experiencing disruption to the normal running of your lab that makes experimental revisions challenging. If it would be helpful, we encourage you to contact us to discuss your revision in greater detail. Please send us a point-by-point response indicating where you are able to address concerns raised (either experimentally or by changes to the text) and where you will not be able to do so within the normal timeframe of a revision. We will then provide further guidance. Please also note that we are happy to extend revision timeframes as necessary.

Please ensure that you clearly highlight all changes made in the revised manuscript. Please avoid using 'Tracked changes' in Word files as these are lost in PDF conversion.

I should be grateful if you would also provide a point-by-point response detailing how you have dealt with the points raised by the reviewers in the 'Response to Reviewers' box. Please attend to

© 2021. Published by The Company of Biologists under the terms of the Creative Commons Attribution License (https://creativecommons.org/licenses/by/4.0/).

all of the reviewers' comments. If you do not agree with any of their criticisms or suggestions please explain clearly why this is so.

Reviewer 1

Advance summary and potential significance to field

SMIFH2 is a small molecule inhibitor of formin FH2 domains. This compound is a widely-used tool for studying the cellular functions of formins with the assumption that it is a specific inhibitor of formins. Here, Nishimura, Shi et al. provide evidence that SMIFH2 also inhibits various myosins in vitro, and consequently affects the contractility of actomyosin bundles in cells. This information will be valuable for the actin cytoskeleton field, because it demonstrates that SMIFH2 is not a specific inhibitor of formins, and hence the results obtained by using SMIFH2 should be carefully analyzed and reconsidered. There are, however, several relatively minor points that should be addressed before publication.

Comments for the author

1. Line 134: The authors claim that Fig. 2B demonstrates that SMIFH2 affected the overall organization of actin and myosin II in cells. However they do not show any actin stainings in the figure, and thus this sentence should be accordingly revised.

2. Line 169: The authors refer to Supplementary figure 1, which demonstrates that SMIFH2 inhibits the basal ATPase activity of myosin II also in the absence of actin filaments. However, this supplementary figure was absent from the manuscript version that I received for review.

3. Lines 175-177: Could the authors discuss a bit more on why the SMIFH2 treatment was irreversible in the actin gliding assay.

4. Figure 1A: Scale bars are missing from the figure.

5. Figure 1C needs more explanation. The authors should discuss why the ends of ventral stress fibers move? Is this because of the phalloidin-treatment and are the stress fibers detached from focal adhesions?

6. Fig. 1C: The authors should indicate in the figure that the time-scale was 30 mins in all kymographs.

7. Figures 3 and 4: Please explain what the error bars represent, and indicate the number of independent experiments ('n') in the figure legend.

8. Table I: Please indicate the number of filaments analyzed.

9. Line 103: authors state that they used 80 uM para-amino Blebbistatin, but based in the figure legend the concentration was 100 uM.

10. Line 106: '….used REF52 permeabilized…' should read '…used REF52 cells permeabilized….'.

11. Line 308: '…necessary exists, since….' should read '…necessary exist since….'.

12. Line 331: Use consistently the 'GFP-MLC' abbreviation for the myosin light chain construct.

Reviewer 2

Advance summary and potential significance to field

A widely used inhibitor of formins seems to also inhibit myosin-II's ATPase.

© 2021. Published by The Company of Biologists under the terms of the Creative Commons Attribution License (https://creativecommons.org/licenses/by/4.0/).

Comments for the author

Many drugs are widely used in cell science but off-target effects not always clear. This submission provides suggestive evidence that a formin inhibitor SMIFH2 also inhibits myosin-2's. Several labs involved all have deep expertise in cytoskeleton, especially contractility extending to purified proteins.

However, a few concerns should be addressed.
1. Fig 3A with purified nonmuscle Myo2A is especially important, but none of the datapoints up to 100uM appears to differ significantly from 0uM. Some indication of pointwise significance is needed relative to 0uM. Also, the equation for the curve fit needs to be written and explained (in each figure), particularly because the curve seems to indicate some type of cooperativity that is not as clear in for the other proteins in Fig 3B and Fig. 4. All of this requires careful explanation, and so does the incomplete inhibition at high drug.
2. Table 1 with purified muscle Myo2 also does not seem to show a significant difference between 0uM and 50uM, at least based on overlapping SD's. Some indication of significance is needed.
3. The two curves in Fig 4A need explanation.
4. Does combining pAB and SMIFH2 at intermediate doses have additive effects?

Reviewer 3

Advance summary and potential significance to field

Highly selective and potent small molecule inhibitors have proven to be invaluable tools for studying the functions of cytoskeletal proteins (e.g. latrunculin A). The development of the mDia1 and mDia2 formin inhibitor SMIFH2 over a decade ago contributed to the characterization of these actin regulators in cytokinesis, actin organization, for example. However, the general electrophilic nature SMIFH2 raised questions about its specificity. Nishimura et al reveal that SMIFH2, quite shockingly, also inhibits members of the myosin superfamily - non-muscle Myo2A, skeletal Myo2, Myo5, Myo7a and Myo10. SHMIFH2 inhibits the actin activated ATPase of Myo5 most potently, with an even higher IC50 than formin itself (~ 2 ÂµM). The in vitro motility of skeletal Myo2 is inhibited, but only at high concentrations of the inhibitor (150 ÂµM). This is not surprising because even if the activity of some myosin motors is inhibited the remaining functional motors can still drive motility. Based on the nature of the inhibition observed, the authors conclude that SMIFH2 inhibits Pi release and blocks myosin(s) in its weak actin-binding state, similar to the widely used myosin inhibitor blebbistatin.

The clear results and careful analysis presented here, a strong combination of cellular and in vitro tests provide convincing support for the conclusion that SMIFH2 is not a specific formin inhibitor. The findings also suggest the possibility that SMIFH2 could have others, as yet unidentified, cellular targets as there is no reason to think that myosins are the only other target for this compound. Based on the results presented here, surely many groups will need to revisit their conclusions or models based in large part on inhibition by SMIFH2. Given the widespread use of SMIFH2 in the cytoskeletal field, this work is significant, timely and represents an important caution to the field about careful assessment and validation of inhibitors.

Comments for the author

1. It is a bit puzzling to find that the authors test the impact of SMIFH2 on the in vitro motility of skeletal Myo2 instead of non-muscle Myo2A given SMIFH2 is mainly used in non-muscle cells. The authors find that skeletal Myo2 is more sensitive to SMIFH2, with an IC50 in the 40 µM range versus 100 µM for non-muscle Myo2A (Fig 3) so one can guess that this was done because skeletal Myo2 is more sensitive to inhibition, but perhaps not. The authors should explain why they chose to show the potential inhibition of skeletal Myo2 motility in vitro instead of the non-muscle Myo2A. (Although it should be noted that in the Discussion the state that 'the gliding of actin filament by myosin 2A in vitro was also suppressed by SMIFH2' - lines 254,255 - no data or details are provided)

2. The initial experiments with cells (Fig 1) show that SMIFH2 most certainly inhibits stress fiber contractility.

© 2021. Published by The Company of Biologists under the terms of the Creative Commons Attribution License (https://creativecommons.org/licenses/by/4.0/).

However, the range of concentrations routinely used to treat cells (40 - 50 μM) has little impact on the enzymatic activity of non-muscle Myo2A (25 - 50 μM; Fig 3A). Could the authors comment on the likelihood of 'off-target' effects of SMIFH2 could really be due to inhibition of non-muscle Myo2A? Have they also tested the other major non-muscle Myo2, Myo2B to see if it is more potently inhibited than Myo2A?

3. The authors show that SMIFH2 does not inhibit MLCK, the key kinase that activates non-muscle Myo2 (Supp Table 2). The way this experiment is introduced (lines 161 - 164) could be a bit confusing to the reader who may not be aware that a key step in the ATPase assay method is pre-incubation of the myosin with MLCK to phosphorylate the regulatory light chain. The authors should try to explain why they thought it important to test SMIFH2 on MLCK more clearly.

4. A table summarizing the IC50 of SMIFH2 compared to that of the myosins tested here would provide readers with an easy recap of the impact of SMIFH2 on these motors.

4. The abstract states that "...inhibition of myosin 2A in vitro required somewhat higher concentrations of SMIFH2..."
This seems unnecessarily vague and the authors should be encouraged to be more specific here.

5. Was a buffer included in the in vitro motility assay solution (line 425)?

First revision

<u>Author response to reviewers' comments</u>

Rebuttal to the comments:

Reviewer 1

Comments for the author
1. Line 134: The authors claim that Fig. 2B demonstrates that SMIFH2 affected the overall organization of actin and myosin II in cells. However, they do not show any actin stainings in the figure, and thus this sentence should be accordingly revised.

We thank the reviewer for pointing this out. Since the ventral actin structures are completely in line with what is expected and follow the myosin pattern, we removed the mention of actin in the said sentence.

2. Line 169: The authors refer to Supplementary figure 1, which demonstrates that SMIFH2 inhibits the basal ATPase activity of myosin II also in the absence of actin filaments. However, this supplementary figure was absent from the manuscript version that I received for review.

We have removed this figure and described the data in the Results section.

3. Lines 175-177: Could the authors discuss a bit more on why the SMIFH2 treatment was irreversible in the actin gliding assay.

There could be several reasons why this is occurring. First, it is possible that, given the high electrophilicity of SMIFH2, the compound is covalently modifying the myosin. Second, the dissociation rate of SMIFH2 from myosin could be slow, but this not so likely given its relatively weak affinity for this myosin. We have added some text in the manuscript to comment on this.

4. Figure 1A: Scale bars are missing from the figure.
Done

© 2021. Published by The Company of Biologists under the terms of the Creative Commons Attribution License (https://creativecommons.org/licenses/by/4.0/).

5. Figure 1C needs more explanation. The authors should discuss why the ends of ventral stress fibers move? Is this because of the phalloidin-treatment and are the stress fibers detached from focal adhesions?

Phalloidin was added to the permeabilization buffer in order to both stabilize and label the actin structures, in particular, stress fibers. We checked that phalloidin treatment alone did not incur and shortening of stress fibers. The retraction of the fibers occurred only upon addition of ATP . It resulted to both centripetal shift of photoconverted actin spots and retraction of the distal ends of the stress fiber corresponding to the focal adhesion plaque.
Since we did not have specific focal adhesion markers in this experiment, we do not know whether contraction of the stress fibers was accompanied by their detachment from focal adhesions, or focal adhesions slid centripetally, driven by contracting stress fibers. We do not think that this question is critically important in context of our study.

6. Fig. 1C: The authors should indicate in the figure that the time-scale was 30 mins in all kymographs.
Done

7. Figures 3 and 4: Please explain what the error bars represent, and indicate the number of independent experiments ('n') in the figure legend.

Error bars are standard deviation. Where error bars are given it represents a minimum of three repetitions. This information has been added to the figure legend.

8. Table I: Please indicate the number of filaments analyzed.

This information has now been included in Table 1. In addition, please note that we analyzed more filaments to increase the n value. Note that we also created a new table (Table 2) that describes the effect of SMIFH2 on the in vitro motility of non-muscle myosin 2A.

9. Line 103: authors state that they used 80 uM para-amino Blebbistatin, but based in the figure legend the concentration was 100 uM.

Thanks for noticing that. We corrected it now. The concentration used was 100 uM.

10. Line 106: '….used REF52 permeabilized…' should read '…used REF52 cells permeabilized….'.
Thank you. Corrected

11. Line 308: '…necessary exists, since….' should read '…necessary exist, since….'.
Thank you. Corrected

12. Line 331: Use consistently the 'GFP-MLC' abbreviation for the myosin light chain construct.

Now we use the abbreviation 'GFP-MRLC' for the myosin II regulatory light chain.

Reviewer 2 Comments for the author

1. Fig 3A with purified nonmuscle Myo2A is especially important, but none of the datapoints up to 100uM appears to differ significantly from 0uM. Some indication of pointwise significance is needed relative to 0uM. Also, the equation for the curve fit needs to be written and explained (in each figure), particularly because the curve seems to indicate some type of cooperativity that is not as clear in for the other proteins in Fig 3B and Fig. 4. All of this requires careful explanation, and so does the incomplete inhibition at high drug.

We conducted a new set of experiments using a higher concentration of non-muscle myosin 2A in order to obtain better quality data for this. We have now included information on the statistical significance of the inhibition for this new graph. The p values for the data from 5 - 20 uM were not statistically different from the control ($p \geq 0.05$) whereas the data points at 50, 75, 100 and

© 2021. Published by The Company of Biologists under the terms of the Creative Commons Attribution License (https://creativecommons.org/licenses/by/4.0/).

200 uM were. (p<0.001). We removed the lines on the graphs that were merely guide for the eyes. We understand that they could be misleading and taken as fits.
We tried to do hyperbolic fits to the data which is what would normally be used for simple reversible inhibition, but did not obtain good fits in all cases. If the inhibition were more complicated such as if covalent modifications were occurring or if there are multiple allosteric binding sites for SMIFH2 on the myosins, then a simple hyperbola would not necessarily fit.

2. Table 1 with purified muscle Myo2 also does not seem to show a significant difference between 0uM and 50uM, at least based on overlapping SD's. Some indication of significance is needed.

We have analyzed more data to increase the n value for this experiment and have replaced the original Table 1 with this. We also expressed the error of the in vitro motility data in terms of standard error which is now standard practice within the field. We have also carried out a statistical analysis of the data and included this in the Tables.

3. The two curves in Fig 4A need explanation.

These were duplicate measurements as now stated in the caption.

4. Does combining pAB and SMIFH2 at intermediate doses have additive effects?

We did not include in this paper the data on combine effect of pAB and SMIFH2. In our recent experiments, which we would like to publish elsewhere, we have demonstrated that addition of pAB inhibits the myosin flow rate in the live cells treated with SMIFH2. This is in agreement with our conclusion that the effect of SMIFH2 on myosin 2A was relatively weak.

Reviewer 3:
1. It is a bit puzzling to find that the authors test the impact of SMIFH2 on the in vitro motility of skeletal Myo2 instead of non-muscle Myo2A given SMIFH2 is mainly used in non-muscle cells. The authors find that skeletal Myo2 is more sensitive to SMIFH2, with an IC50 in the 40 µM range versus 100 µM for non-muscle Myo2A (Fig 3) so one can guess that this was done because skeletal Myo2 is more sensitive to inhibition, but perhaps not. The authors should explain why they chose to show the potential inhibition of skeletal Myo2 motility in vitro instead of the non-muscle Myo2A.

(Although it should be noted that in the Discussion the state that 'the gliding of actin filament by myosin 2A in vitro was also suppressed by SMIFH2' - lines 254,255 - no data or details are provided)

The reviewer is correct that we chose to present the data of the effect of SMIFH2 on the in vitro motility of skeletal muscle myosin 2 since the drug more potently inhibited this myosin, but we have now presented data on the inhibition of non-muscle Myo2A in a new table (Table 2). Please note, as we discussed in the Discussion, that the in vitro motility assay is not a particularly good assay to study titrations of inhibitors of myosins. If the inhibitor blocks the myosin in a weakly actin binding state (as does blebbistatin, for instance), then the extent of inhibition will not parallel that of the inhibition of the ATPase activity. This is due to the fact that the rate of movement of actin filaments does not strongly depend on the number of myosin motors acting on the filament. Therefore, inhibition of 50% of the motors will not inhibit the rate of actin filament movement by 50%.

2. The initial experiments with cells (Fig 1) show that SMIFH2 most certainly inhibits stress fiber contractility. However, the range of concentrations routinely used to treat cells (40 - 50 µM) has little impact on the enzymatic activity of non-muscle Myo2A (25 - 50 µM; Fig 3A). Could the authors comment on the likelihood of 'off-target' effects of SMIFH2 could really be due to inhibition of non-muscle Myo2A? Have they also tested the other major non-muscle Myo2, Myo2B to see if it is more potently inhibited than Myo2A?

We tried very hard to quantify the inhibition of non-muscle Myo2B by SMIFH2, but were unsuccessful in getting reproducible data. Part of this was due to the very low ATPase activity of this myosin (3X slower than is non-muscle Myo2A) and that we had only limited amounts of this myosin available. Also, we only had full length non-muscle Myo2B available for assays. It is very

© 2021. Published by The Company of Biologists under the terms of the Creative Commons Attribution License (https://creativecommons.org/licenses/by/4.0/).

difficult to obtain linear time courses of activity in our spectrophotometric assay given light scattering artifacts that arise when myosin filaments bind to actin filaments and create contractile networks which float in and out of the light beam. We did not have any HMM fragments of this myosin available and were unable to produce them in time for this revision given the restrictions the Sellers' lab is operating under now due to covid-19.

As we indicated in the paper and in the answer to the reviewer 2, the effect of SMIFH2 on myosin 2A was the weakest as compared to the effects on other myosins. Therefore, we do not think that effect of SMIFH2 on cell contractility is entirely due to its effect on myosin 2A. As we hypothesize in the discussion, the SMIFH2 induced detachment of actin filaments from formin which most probably decreases actin cytoskeleton connectivity could be the major mechanism of SMIFH2 driven contractility inhibition.

3. The authors show that SMIFH2 does not inhibit MLCK, the key kinase that activates non-muscle Myo2 (Supp Table 2). The way this experiment is introduced (lines 161 - 164) could be a bit confusing to the reader who may not be aware that a key step in the ATPase assay method is pre-incubation of the myosin with MLCK to phosphorylate the regulatory light chain. The authors should try to explain why they thought it important to test SMIFH2 on MLCK more clearly.

We have rewritten this.

4. A table summarizing the IC50 of SMIFH2 compared to that of the myosins tested here would provide
readers with an easy recap of the impact of SMIFH2 on these motors.

We have created a new Table of approximate IC50 values. Please realize that ,in the absence of a good curve fit, these will be approximations.

4. The abstract states that "…inhibition of myosin 2A in vitro required somewhat higher concentrations of SMIFH2…"
This seems unnecessarily vague and the authors should be encouraged to be more specific here.

We have rewritten this to remove "somewhat".

5. Was a buffer included in the in vitro motility assay solution (line 425)?

We apologize for the omission. This is now included in the Materials and Methods.

Second decision letter

MS ID#: JOCES/2020/253708

MS TITLE: The Formin Inhibitor, SMIFH2, Inhibits Members of the Myosin Superfamily

AUTHORS: Yukako Nishimura, Shidong Shi, Fang Zhang, Rong Liu, Yasuharu Takagi, Alexander D Bershadsky, virgile viasnoff, and James R Sellers
ARTICLE TYPE: Research Article

I am happy to tell you that your manuscript has been accepted for publication in Journal of Cell Science, pending standard ethics checks.

© 2021. Published by The Company of Biologists under the terms of the Creative Commons Attribution License (https://creativecommons.org/licenses/by/4.0/).
